# Infectious Complications of DiGeorge Syndrome in the Setting of Malignancy

**DOI:** 10.7759/cureus.26277

**Published:** 2022-06-24

**Authors:** Heather Hare, Pragya Tiwari, Aliyah Baluch, John Greene

**Affiliations:** 1 College of Pharmacy, University of Florida Health, Tampa, USA; 2 Cardiology, Hridaya Clinic, Delhi, IND; 3 Infectious Diseases, Moffitt Cancer Center, Tampa, USA; 4 Internal Medicine, Moffitt Cancer Center, Tampa, USA

**Keywords:** lymphopenia, t-cell deficiency, hodgkin’s lymphoma, infectious disease, immunodeficiency, 22q11.2 deletion syndrome, digeorge syndrome

## Abstract

This report describes a case of a young man with DiGeorge Syndrome, repaired Tetralogy of Fallot, relapsed metastatic Hodgkin’s Lymphoma, immunodeficiency, and a history of recurrent and severe infections. A review of the literature indicates that patients with DiGeorge Syndrome are at greater risk for infection, malignancy, and cardiac events due to anatomic and immunologic complications resulting from a deletion in the 22q11.2 chromosome. As an increased number of patients with DiGeorge Syndrome are surviving into adulthood, it is important to understand the progression of the disease and the long-term implications associated with variable degrees of thymic hypoplasia and immune deficiency.

## Introduction

DiGeorge Syndrome (DGS) is a genetic disorder resulting from a microdeletion on the long arm of chromosome 22 at the locus 22q11.2 [1]. This deletion syndrome can be referred to by a variety of additional names including chromosome 22q11.2 deletion syndrome, velocardiofacial syndrome, Cayler cardiofacial syndrome, Shprintzen syndrome, and Catch-22 syndrome, which represent the same collection of findings based on genetic testing [1,2]. DGS is one of the most common chromosome deletion syndromes with an incidence of approximately 1 in 3000 to 4000 births with 90% of these being the result of de novo events [1,3,4]. The absence of the thymus, known as complete DGS, is a rare complication of 22q11.2 deletion representing approximately 1.5% of cases compared to those with partial DGS [5,6]. The deletion itself most commonly consists of a 3.5Mb region on chromosome 22, but a smaller subset of patients have been found to have a 1.5Mb deletion which does not correlate with a milder phenotype [1,4]. Major characteristics of DGS include cardiac malformation, palatal anomalies, and thymic hypoplasia with associated hypocalcemia, hypoparathyroidism, and immunodeficiency [2]. Both immunologic and anatomic complications lead to an increased risk for recurrent infections and autoimmunity [5]. Additionally, many patients suffer from developmental delay, psychiatric illness, and behavioral challenges which become increasingly challenging in the transition to adulthood [7]. Improved diagnostics and early intervention in children with DGS have led to an increase in those surviving to adulthood and thus a greater need for understanding the long-term clinical implications [1,3].

## Case presentation

The patient is a 30-year-old male with a past medical history of DiGeorge Syndrome, Tetralogy of Fallot surgically repaired at three years old, and relapsed stage IV Hodgkin’s Lymphoma. The patient was initially diagnosed with Epstein-Barr virus-induced lymphoma at the age of 24. Since diagnosis, the patient’s lymphoma treatment has consisted of a splenectomy due to unresponsive pancytopenia, four cycles of doxorubicin, bleomycin, vinblastine, dacarbazine (ABVD) treatment, six cycles of single-agent Brentuximab, fourteen doses of Nivolumab, and one cycle of ifosfamide, carboplatin, etoposide (ICE) treatment which was complicated by fevers and tachycardia and thus discontinued. The patient has known immunodeficiency including T-cell deficiency, lymphopenia, and hypogammaglobulinemia. Recent immunoglobulin levels included IgA <5 mg/dL, IgM 13 mg/dL, IgG was 762 mg/dL. Additionally, he had low T-lymphocytes, B-lymphocytes, and Natural Killer cell counts, as shown in Table 1. During childhood, the patient developed multiple bacterial infections requiring five myringotomies for ear infections, three sinusotomies for sinusitis, and an adenoidectomy. He also experienced recurrent respiratory and fungal infections including one episode of pneumocystis pneumonia requiring tracheostomy at age 25 for which he requires lifelong antibiotic secondary prophylaxis. The frequency of pneumonia improved after starting Intravenous Immunoglobulins (IVIG) infusions at age 26. Infectious disease history in the two years prior to consultation includes a port-associated Staphylococcus epidermidis infection, oral candidiasis, Escherichia coli bacteremia, disseminated varicella-zoster, pneumonia complicated by bilateral pleural effusions, and severe Clostridium difficile associated colitis complicated by a gastrointestinal hemorrhage requiring an ICU admission. He also contracted COVID-19 for a month in December of 2020 and was hospitalized at that time.

**Table 1:** Laboratory Values Lab results from July 2021 occurred just prior to the patient's intravenous immunoglobulin infusion. Comparative values are shown from December 2021 and February 2022.

Laboratory Test	July 2021	December 2021	February 2022
IgG (mg/dL)	782	458	---
IgA (mg/dL)	<5	---	---
IgM (mg/dL)	13	---	---
Lymphocytes, abs (k/uL)	0.2	0.12	0.16
CD3 Total T Cells (k/uL)	---	---	0.144
CD4 Helper/Inducer (k/uL)	---	---	0.014
CD8 Suppressor/Cytotoxic (k/uL)	---	---	0.127
CD19 Total B Cells (k/uL)	---	---	0.005
CD16+/CD56+ NK Cells (k/uL)	---	---	0.096
Ferritin (ng/dL)	992	331	3298
Interlukin 2 Receptor (CD25) (pg/mL)	22770	3701	33855
Triglycerides (mg/dL)	101	107	102
Epstein-Barr Viral Load (IU/mL)	2,907	13,225	191,036

The patient was treated at an outside hospital two months prior to consultation and found to have fluid overload due to systolic heart failure for which he was started on a diuretic and a beta-blocker. An echocardiogram revealed an ejection fraction of 46%. The patient also has a significant history of atrial fibrillation and a prior deep vein thrombosis of the right iliac vein. 

**Figure 1:**
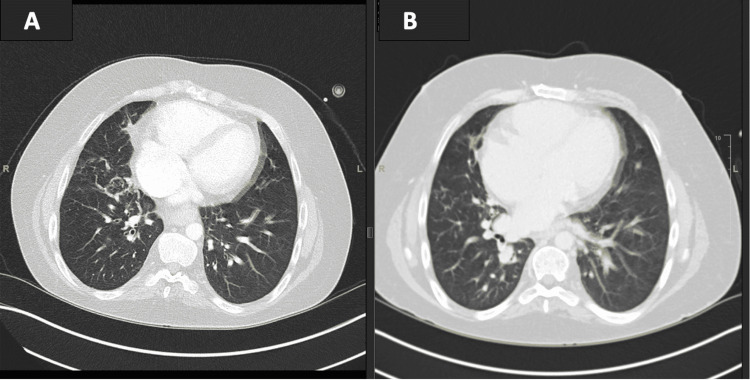
CT Chest with Comparison Case imaging shows bronchiectasis and scarring of the lung bases from prior pulmonary infections during childhood and young adulthood. Figure 1A is from May 2020. Figure 1B is from July 2021.

The patient presented to Moffitt Cancer Center with three weeks of persistent fevers up to 102°F, suspected to be attributed to his malignancy, as well as oxygen desaturation to 90-92% per home pulse oximetry. He was accompanied by his mother who reported that the patient was also experiencing worsened fatigued and a chronic cough. Initial vitals upon admission revealed that the patient had a temperature of 99.4 F, a heart rate of 107, and low blood pressure at 88/53. His oxygen saturation was 96% on 2 liters of oxygen via nasal cannula. Physical examination revealed bibasilar crackles and tachycardia. Initial labs and blood cultures were negative for any significant abnormalities. The computed tomography (CT) scan without contrast of the chest (shown in Figure 1B) showed bronchiectasis and scarring of the lung bases presumed to be from numerous pulmonary infections during childhood and young adulthood. This CT scan can be compared to imaging from May of 2020, one year earlier (shown in Figure 1A). The patient subsequently reached a maximum temperature of 102.8°F inpatient and became increasingly hypotensive requiring a transfer to the ICU. Cefepime and Levofloxacin were started for atypical pneumonia coverage in addition to the continuation of prophylactic Atovaquone. A fiber-optic bronchoscopy with bronchoalveolar lavage was performed and initial cultures were unremarkable. Additionally, a bone marrow biopsy was performed to evaluate for hemophagocytic lymphohistiocytosis (HLH). Bone marrow biopsy revealed focal hemophagocytic activity, specifically erythrophagocytosis, which was non-specific and not characteristic of HLH. Considering the setting of abnormal lab values as shown in Table 1, and a history of splenectomy, HLH became more of a concern. Additionally, as compared to one month prior, the patient’s Epstein-Barr viral load increased substantially from 13,255 IU/mL to 191,036 IU/mL. The patient was empirically started on high-dose dexamethasone. 

The patient’s extensive workup revealed no evidence of acute infectious processes. Given his decline in health due to refractory Hodgkin’s lymphoma, the family elected to move forward with hospice care and discontinued all medications except acetaminophen and prednisone for comfort measures. The patient passed away just eight days later in inpatient hospice care.

## Discussion

Patients with DiGeorge Syndrome have impaired development or absence of the thymus leading to immunodeficiency and increased susceptibility to severe and recurrent infections [5,6]. Due to varying degrees of thymic hypoplasia, patients fall along a spectrum with phenotypes ranging from normal to severe T-lymphopenia [8]. Overall, patients with DGS experience an increased incidence of immune dysfunction and autoimmunity compared to the general population [7]. Studies have shown an association between the magnitude of immune deficiency and the location of the deletion in chromosome 22q11.2 [8]. Deletions that include the TBX1 gene appear to result in more marked immunodeficiency compared to other distal deletions [8]. A study by Crowley et al. of lymphocyte counts in 52 infants showed that cluster of differentiation (CD)3 and CD4 counts were significantly reduced in subjects with a TBX1 deletion compared to those without this deletion [8]. Oppositely, CD8, CD19, and natural killer (NK) counts were unaffected by deletion at the TBX1 locus suggesting that additional genes in the deleted region are responsible [8]. Our patient had low counts of all lymphocytes which speaks to the role of deletion breakpoints in the determination of phenotype. T-cell deficiency in childhood best reflects the degree of thymic hypoplasia but is typically corrected by adulthood through secretion of IL-7 stimulating T-lymphocyte proliferation and resulting in a normal appearance of the lymphocyte counts [8]. Furthermore, homeostatic expansion has an impact on humoral immunity through reduced aid of B-cells despite apparent normal peripheral T-cell levels [8]. This results in many adult patients developing immunoglobulin deficiency, particularly IgM deficiency, impairing their ability to fight off bacterial infections, particularly gram-negative bacteria [8]. A study by Giardino et al. assessing clinical features of DGS in a group of 447 patients noted autoimmunity in 7.8% of subjects and reported that nine out of these 35 cases of autoimmunity were due to autoimmune cytopenia [5]. There were statistically significant relationships between autoimmunity and IgM deficiency as well as between autoimmunity and lymphopenia in the population studied [5]. Other common autoimmune manifestations include juvenile idiopathic arthritis, idiopathic thrombocytopenia purpura (ITP), and autoimmune hemolytic anemia [4,6]. Furthermore, long-term impaired immunity, infections, and malignancy may precipitate hemophagocytic syndromes which result in life-threatening inflammation [9].

Common anatomic abnormalities including cardiac, respiratory, ear-nose-throat (ENT), and gastrointestinal anomalies lead to a greater risk for chronic inflammation and frequent or severe infections in multiple organ systems [5]. Giardino et al. reported recurrent or severe infections in 64.4% of subjects [5]. Multivariate logistic regression analysis by the researchers determined that the increase in recurrent infections was associated with anatomic changes [5]. Specifically, palatal malformations presented a risk for ENT infections, and gastroesophageal reflux, dysphagia, asthma, and rhinitis presented a risk for respiratory infections [5]. Additionally, respiratory tract abnormalities including airway malacia and bronchiectasis from recurrent infections lead to poor pulmonary function and increased morbidity and mortality [5,10]. This was evident in our case, as displayed in the imaging shown in Figure 1.

Complete DGS, characterized by congenital athymia, represents a rare subset of patients with DGS, present in approximately 1.5% of cases [5,6]. This condition is most often fatal in infancy without correction by thymus transplantation or hematopoietic cell transplantation [4]. Individuals with complete DGS are much more prone to severe infectious diseases and infection by opportunistic pathogens. Our review of the literature, summarized in Table 2, showed infection with several additional pathogens including several bacterial, fungal, and viral organisms including Staphylococcus lugdunensis, Aspergillus, and cytomegalovirus. Despite these more severe cases, the most common type of infection in patients with partial DGS remains community-acquired viral respiratory infections [4,6]. 

**Table 2:** Summary of studies related to infectious implications of DiGeorge Syndrome

Study/Year	Age/Sex	Presenting Symptoms	Infection	Pathogen
Bluestone et al. [11]	8/M	13 reoccurrences of infection	Colitis	Clostridium difficile
Lozano-Chiga et al. [12]	26/M	Fever and cough	Pneumonia	Pneumocystis jiroveccii
Chang et al. [13]	14/M	Fevers, fatigue, dyspnea	Type III Mixed Cryobulinemia and Antiphospholipid Syndrome	Streptococcus pyogenes (Group A)
Franciosi et al. [14]	2/M	Melena and peritonitis	Pneumoperitoneum	Aspergillus
Walls et al. [15]	10/F	Abdominal pain, appetite loss, intermittent fevers	Endocarditis	Bartonella henselae
Hirasaki et al. [16]	27/F	Fatigue and fever	Septicemia, likely origin dental infection	Staphylococcus ludgunensis
Yin et al. [17]	1/M	Fever and cough	Pneumonia	Pneumocystis jirovecii, Mycobacterium kansasii
Lewis et al. [18]	23/M	Unspecified	Moderate/Severe COVID-19	SARS-CoV-2 (COVID-19)
Suksawat et al. [19]	Multiple Average 3.5mo	Unspecified	Septicemia, unspecified origin	Cryptocoocus neoformans Candida tropicalis
Deerojanawong et al. [10]	Multiple Average 3mo	Unspecified	Bronchomalacia, recurrent pulmonary infections, atelectasis, lung fibrosis	Escherichia coli, Haemophilus influenzae, Flavibacterium, Respiratory syncytial virus, Adenovirus, Klebsiella pneumoniae, Corynebacterium, Parainfluenza type 3, Cytomegalovirus

Characteristic lymphocyte impairment is also associated with an increased risk of malignancy as seen in our case and the case by Lozano-Chinga et al. [12]. The most common cancers found in primary immunodeficient patients are non-Hodgkin’s lymphoma, Hodgkin’s lymphoma, and acute leukemia [2,20]. A study conducted by McDonald-McGinn et al. examined the risk of malignancy in children with DGS through a multicenter chart review (n=687) [20]. They found that the overall rate of malignancy in children under 14 was approximately 900 out of every 100,000 (0.9%) in the setting of chromosome 22q11.2 deletion compared to only 3.4 out of every 100,000 (0.0034%) in children without DGS [20]. Given the variability in deletion size and location within the 22q11 region, the chromosomal deletion itself may play a role in oncogenesis through DNA damage, instability, and various downstream mutations at loci responsible for cell cycle regulation such as those associated with the RAS-Mitogen Activated Protein Kinase (RAS-MAPK) signaling pathway [2]. Another candidate gene, when included in the deletion, is the catechol-O-methyltransferase (COMT) gene responsible for protection against environmental carcinogens [2,20]. Studies have also shown an association between distal deletions including the SMARCB1 tumor suppressor gene and the incidence of rhabdoid tumors [6]. Other factors include the presence of T-cell deficiency and anatomic abnormalities that exacerbate infectious processes and lead to chronic inflammation [2,5,20]. 

Our case presents some of the major complications of DGS in a single individual including cardiac malformation, malignancy, recurrent infection, and immune dysfunction. Given the medical and social complexity of DGS patients, along with their predisposition for infectious disease and autoimmune conditions, it is crucial for healthcare providers to understand the best practices for managing such patients, especially in adult populations [6]. Additionally, the spectrum of features found in DGS requires extensive collaboration between medical specialties such as cardiology, pulmonology, immunology, oncology, and infectious disease [6]. Although less research has been conducted on the progression of 22q11.2 deletion syndrome in adult patients, a study by Van et al. examining all-cause mortality of 309 adult DGS patients found that 70.1% of deaths were due to cardiac events likely related to long-term effects of cardiac malformations [3]. Other concerns include progressive lymphocyte dysfunction due to homeostatic proliferation, and the increased risk for autoimmunity and malignancy [6]. Some patients, such as the young man in our case, may require prophylactic antibiotics or IVIG treatment for the prevention of infection [4]. Others may benefit from the correction of anatomic abnormalities such as palatal deformities that exacerbate ENT infections [4]. Although guidelines have yet to be established, patients with known T-cell lymphopenia may benefit from closer monitoring of T-cell and immunoglobulin levels depending on the severity of the immunodeficiency and frequency of infections [6]. Overall, closer observation of both pediatric and adult DGS patients is necessary due to susceptibility for serious complications and the wide range of phenotypes that occur as a result of the 22q11.2 deletion [6,7].

## Conclusions

This report highlights some of the potential consequences of the chromosome 22q11.2 deletion syndrome known as DiGeorge Syndrome. As seen in the presented case, such patients are vulnerable to numerous health complications such as cardiac events, malignancy, and severe infectious disease. There is a need for increased vigilance and provider communication in such patients due to the wide range of systems involved and the need for multidisciplinary management. Corrective surgical interventions, infection prevention, and increased knowledge of genetic mechanisms has improved the survival rate and quality of life of DGS patients, but further study remains necessary to support an older population with developing health demands.
